# A Productive Expression Platform Derived from Host-Restricted Eilat Virus: Its Extensive Validation and Novel Strategy

**DOI:** 10.3390/v13040660

**Published:** 2021-04-11

**Authors:** Lu Tan, Yiwen Zhang, Xingxing Wang, Dal Young Kim

**Affiliations:** Department of Infectious Diseases and Public Health, Jockey Club of Veterinary Medicine and Life Sciences, City University of Hong Kong, Kowloon Tong, Hong Kong 999077, China; lutan3-c@my.cityu.edu.hk (L.T.); zhang.yiwen@my.cityu.edu.hk (Y.Z.); xingxing.wang@my.cityu.edu.hk (X.W.)

**Keywords:** Eilat virus, expression platform, insect-specific, diagnostic tool, vaccine candidate

## Abstract

Most alphaviruses are transmitted by mosquitoes and infect a wide range of insects and vertebrates. However, Eilat virus (EILV) is defective for infecting vertebrate cells at multiple levels of the viral life cycle. This host-restriction property renders EILV an attractive expression platform since it is not infectious for vertebrates and therefore provides a highly advantageous safety profile. Here, we investigated the feasibility of versatile EILV-based expression vectors. By replacing the structural genes of EILV with those of other alphaviruses, we generated seven different chimeras. These chimeras were readily rescued in the original mosquito cells and were able to reach high titers, suggesting that EILV is capable of packaging the structural proteins of different lineages. We also explored the ability of EILV to express authentic antigens via double subgenomic (SG) RNA vectors. Four foreign genetic materials of varied length were introduced into the EILV genome, and the expressed heterologous genetic materials were readily detected in the infected cells. By inserting an additional SG promoter into the chimera genome containing the structural genes of Chikungunya virus (CHIKV), we developed a bivalent vaccine candidate against CHIKV and Zika virus. These data demonstrate the outstanding compatibility of the EILV genome. The produced recombinants can be applied to vaccine and diagnostic tool development, but more investigations are required.

## 1. Introduction

The genus *Alphavirus*, in the family of *Togaviridae*, is a group of enveloped viruses that package a positive-strand genomic RNA [1]. This genomic RNA is about 11–12 kb in length and mimics the eukaryotic messenger RNAs (mRNAs). It comprises two open reading frames (ORFs). The first ORF is located at the 5′ region of the genome. Upon arrival in the cytoplasm, it is translated into a nonstructural polyprotein, which forms replication complexes and is responsible for synthesizing genomic and subgenomic (SG) RNAs. The second ORF is located downstream of an SG promoter and encodes the structural proteins, which ultimately package genomic RNA and assemble it into infectious viral particles [1]. The virus-specific RNA and protein synthesis are highly efficient and lead to the ease of producing high-titer viruses. Combined with cDNA clones’ availability and genetic tractability [2,3,4], alphaviruses have been engineered in multiple ways to develop gene expression systems [5,6]. Generally, there are two principal strategies: (i) replacing the ORF encoding structural proteins with the gene of interest to produce self-replicating replicons and (ii) constructing replication and packaging-competent recombinants by introducing an additional SG promoter [6]. Moreover, chimeric alphaviruses, consisting of genetic backbones derived from one alphavirus and structural proteins of another alphavirus, can be generated to develop attenuated alphaviruses [7,8,9,10] and utilized as experimental tools [11,12], providing another strategy to manipulate the alphavirus genome.

The majority of the 31 recognized alphavirus species are transmitted by mosquitoes and infect a wide range of insects and vertebrates [13]. Consequently, alphaviruses can propagate in many insect and vertebrate cell lines. However, there is an exception—Eilat virus (EILV)—which is defective for infecting vertebrate cells due to its inability to enter the cell and replicate its genome in the cytoplasm [12,14,15], albeit the details remain unclear. On the other hand, EILV can replicate to high titers in mosquito cells [14]. This host-restricted character provides a highly advantageous safety profile and renders the EILV–mosquito cell combo an attractive system for producing heterologous proteins. The expressed proteins are either incorporated into the virus particles or released individually, depending on the construction strategies and the protein properties, and can be utilized for vaccination and diagnosis. The safety-guaranteed biosafety level 1 property also makes the production of EILV and EILV-based recombinant viruses much easier and more economical than that of other live viruses, which may require appropriate biocontainment infrastructure, training, and safety protocols. In addition, the absence of chemical or physical inactivation allows the expressed heterologous proteins to maintain wild-type antigenicity and immunogenicity [16]. Compared with other insect-based expression platforms, such as the well-established baculovirus system [17,18], EILV-based vectors bypass the laborious construction process by directly inserting the gene of interest into the EILV cDNA sequence and, moreover, can load expressed heterologous proteins into virus particles in some cases.

EILV-based chimeric virus has been generated using the backbone of EILV and the structural genes of Chikungunya virus (CHIKV). It was utilized as an antigen for CHIKV serological diagnosis, leading to the sensitive detection of CHIKV infection and outperforming traditional antigen preparations [19]. Furthermore, it was developed as a highly immunogenic vaccine for Chikungunya fever, showing protective efficacy in multiple animal models after a single dose [16]. Another two chimeras containing the structural proteins of Venezuelan equine encephalitis virus (VEEV) or Eastern equine encephalitis virus (EEEV) were also investigated as potential vaccine candidates [20]. These different chimeras suggested that EILV can tolerate the structural proteins of other alphaviruses. However, it is implausible to define the tolerability of the EILV genome with a limited number of chimeras. Besides, these reported chimeras exhibited different fitness changes in vitro. EILV/EEEV yielded a reduced rescue titer, while EILV/CHIKV and EILV/VEEV produced comparable or higher titers than EILV [16,20]. Characterization of more EILV-based chimeras may shed light on the underlying reasons for this difference.

Alphavirus-derived double SG vectors are conventionally utilized to express reporter proteins [21]. The sequence of enhanced red fluorescent protein (eRFP) has been introduced into the EILV genome to measure virus replication [12,14,20,22]. There are also EILV/VEEV chimeras expressing green fluorescent protein (GFP) or nanoLuc, used as unique experimental tools [23]. However, the feasibility of exploiting the EILV-based double SG vector to produce authentic antigens has not been investigated. Unlike the small size of reporter genes, the length of viral pathogen-derived antigens can be large. Although one Japanese encephalitis virus antigen cassette, as long as 3.2 kb, has been reported to be inserted into the Sindbis virus (SINV) genome [24], the double SG vectors are primarily useful for heterologous sequences shorter than 2 kb [6]. The ability of the EILV genome to encompass inserts of varied length needs to be determined. Moreover, some antigens require post-translational modifications to increase solubility and enhance antigenicity and virulence properties [25]. Whether the EILV-based double SG recombinant system can accurately confer such modifications remains to be determined.

In the present study, we assessed the feasibility of engineering the EILV genome to generate versatile expression platforms. First, we constructed a series of chimeras containing the structural proteins of genetically distant alphaviruses individually and compared their replication kinetics with the parental EILV. We also utilized EILV-based double SG vectors to produce heterologous antigen proteins recognized by antigen-specific antibodies. By introducing an additional SG promoter into the EILV/CHIKV chimera, we then developed a bivalent vaccine candidate against CHIKV and Zika virus (ZIKV). The CHIKV and ZIKV antigens were detected in the infected cells simultaneously. Our study demonstrated the compatibility of the EILV genome. These EILV-based expression platforms hold great promise to be applied to the development of various vaccines and diagnostic tools.

## 2. Materials and Methods

### 2.1. Cells and Viruses

*Aedes albopictus* C7/10 cell was provided by Professor Scott Weaver, University of Texas Medical Branch, Galveston, USA. Cells were cultivated at 28 °C with 5% CO_2_ in Dulbecco’s Modified Eagle Medium (DMEM) containing 10% heat-inactivated fetal bovine serum (FBS), 1% tryptose phosphate broth, 2 mM L-glutamine, 100 U/mL penicillin, and 100 μg/mL streptomycin. *Cercopithecus aethiops* kidney epithelial Vero E6 cell was obtained from American Type Culture Collection (ATCC), Manassas, VA, USA. Cells were maintained at 37 °C with 5% CO_2_ in Minimum Essential Medium (MEM) alpha supplemented with 10% FBS, 2 mM L-glutamine, vitamins, and penicillin–streptomycin.

The full-length cDNA clone of EILV was provided by Professor Scott Weaver. The full-length cDNA clone of SINV strain Toto 1101 was obtained from Professor Charles Rice’s lab, The Rockefeller University, USA.

### 2.2. Plasmid Constructs

The plasmids encoding EILV-based chimeric alphaviruses were constructed by replacing the structural genes of EILV (GenBank: JX678730.1) with those of SINV strain Toto 1101, Mayaro virus (MAYV) strain Acre27 (GenBank: KM400591.1), o’nyong’nyong virus (ONNV) strain SG650 (GenBank: AF079456.1), CHIKV strain Caribbean (GenBank: LN898112.1), VEEV strain TC83 (GenBank: L01443.1), Western equine encephalitis virus (WEEV) strain McMillan (GenBank: GQ287640.1), or EEEV strain FL93-939 (GenBank: EF151502.1), respectively. The structural genes of MAYV, ONNV, CHIKV, VEEV, WEEV, and EEEV were obtained from a gene synthesis service (GenScript, Piscataway, NJ, USA). EILV-based double SG RNA vectors were created by introducing an additional SG promoter into the EILV genome upstream of the structural genes. The cDNA sequence of non-structural 1 (NS1) protein from ZIKV strain H/PF/2013 (GenBank: KJ776791.2) was acquired from GenScript. African swine fever virus (ASFV) p17, p30, and p72 sequences were derived from ASFV isolate Georgia 2007/1 (GenBank: FR682468.1) and purchased from a gene synthesis service (Integrated DNA Technologies, Coralville, IA, USA). EILV/CHIKV chimera expressing ZIKV pre-membrane and envelope (prME) proteins was generated by introducing an additional EILV subgenomic promoter upstream of the CHIKV structural genes. Two ZIKV prME sequences, from either Asian strain H/PF/2013 (GenBank: KJ776791.2) or African strain MR766 (GenBank: MK105975.1), were adopted and chemically synthesized by GenScript. Standard polymerase chain reaction (PCR) techniques and recombinant DNA techniques were applied to generate these plasmid constructs [12,16,20]. The sequence information is available upon request.

### 2.3. Recombinant Virus Rescue

The plasmids constructed above were sequenced and then purified by QIAGEN Plasmid Maxi Kit, linearized by digestion with NotI (New England Biolabs, Ipswich, MA, USA), and purified by phenol–chloroform extraction. Capped RNAs were then synthesized in an in vitro transcription system containing linearized DNA template, NTP mix, dithiothreitol, RNaseOUT ribonuclease inhibitor, SP6 RNA polymerase (Thermo Scientific, Waltham, MA, USA), and G(5′)ppp(5′)G RNA cap structure analog (New England Biolabs, MA, USA). After being analyzed by agarose gel electrophoresis, these RNAs were electroporated into C7/10 cells without further purification. The EILV-based chimeras were collected at 48 h post-electroporation (hpe), and the EILV-based double SG RNA vectors were collected at 72 hpe. The titers were measured by performing plaque assays on C7/10 cells as previously described [12,14].

### 2.4. Analysis of Virus Replication

C7/10 or Vero E6 cells were seeded into 6-well plates at a density of 5 × 10^5^ cells/well and inoculated with viruses with the indicated multiplicities of infections (MOIs). After one-hour incubation at 28 °C or 37 °C, the cells were washed with phosphate-buffered saline (PBS) three times and covered with 1 mL complete DMEM or MEM alpha medium, followed by further incubation. Cell supernatant was harvested at the designated post-infection time points and replaced with fresh medium. The titers of the collected samples were measured by plaque assay on C7/10 cells [12,14].

### 2.5. Immunofluorescence Assay (IFA)

C7/10 cells were seeded into 24-well plates at a density of 3 × 10^5^ cells/well. Then, 50 µL of pre-diluted viruses was added to cells with an MOI of 5. Following one-hour incubation, 0.5 mL of complete medium was added to each well without removing the inoculum. Cells were incubated for 24 h, the overlay was removed, and the monolayers were fixed with pre-chilled methanol. Cells were then permeabilized with 0.5% Triton X-100 in PBS and stained with primary and secondary antibodies (Table 1). The fluorescence was visualized on a Nikon ECLIPSE Ti2 microscope.

### 2.6. Statistical Analysis

To compare the titers of the parental EILV and the recombinant viruses at each time point of the growth curves, an ordinary 2-way ANOVA using Dunnett’s multiple comparisons test or Šidák’s multiple comparisons test was performed. To compare the optimal titers obtained by each virus with two different MOIs, an ordinary 2-way ANOVA using Šidák’s multiple comparisons test was performed. To compare the optimal titers reached by the parental EILV and the recombinant viruses with the same MOI, an ordinary 2-way ANOVA using Dunnett’s multiple comparisons test or Šidák’s multiple comparisons test was performed. To compare the optimal titers obtained by EILV, EILV/CHIKV, and two chimeric double SG vectors, an ordinary one-way ANOVA using Tukey’s multiple comparisons test was performed. All statistical calculations were performed in Prism 9 (GraphPad Software, San Diego, CA, USA).

## 3. Results

### 3.1. Construction of EILV-Based Chimeras

**Figure 1:**
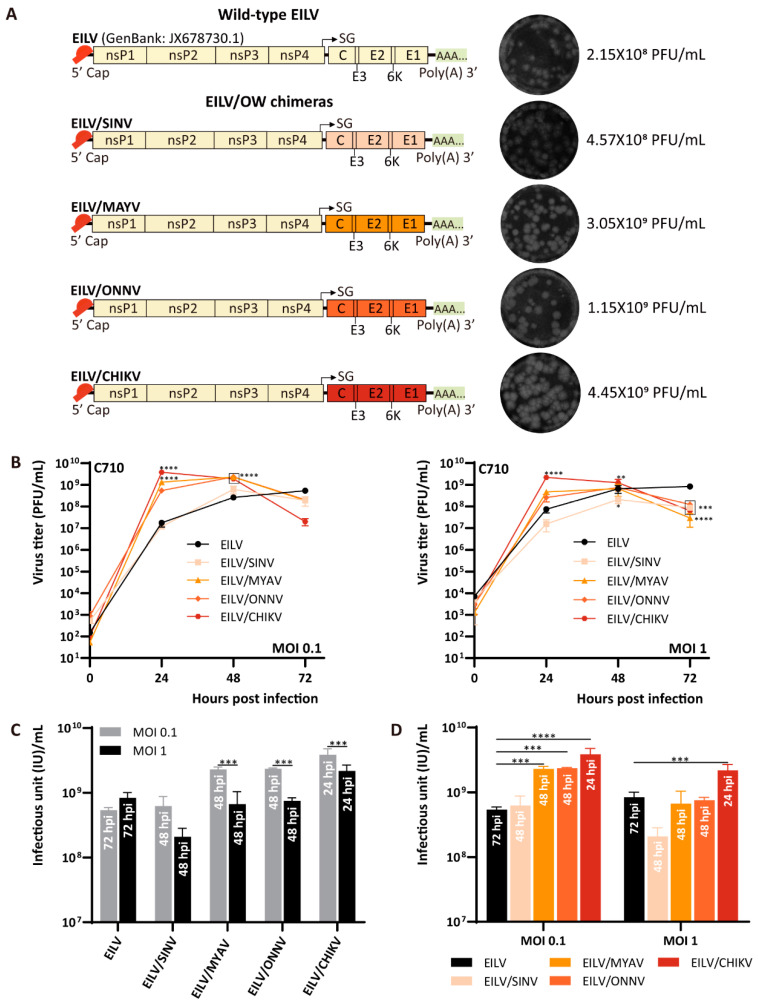
Genome organization and replication kinetics of Eilat virus/ Old World (EILV/OW) chimeras. (**A**) Genome organization of EILV/OW chimeras. Viruses were rescued on C7/10 cells and harvested at 2 days post-electroporation (dpe). Rescue titers and plaque phenotypes are displayed. (**B**) Replication of EILV/OW chimeras in C7/10 cells. Cells were infected at a multiplicity of infection (MOI) of 0.1 or 1. Media were collected at the indicated time points post-infection, and titers were determined by plaque assay. (**C**) The optimal titers obtained by each virus with different MOIs. (**D**) Comparison of the optimal titers produced by EILV and EILV/OW chimeras. The annotation embedded in each bar in (**C,D**) indicates the time point of reaching the highest titer. Each sample was titrated twice (*n* = 2). Average titers ± standard deviations (SD) (error bars) are shown. *, *p* < 0.05; **, *p* < 0.01; ***, *p* < 0.001; ****, *p* < 0.0001.

**Figure 2:**
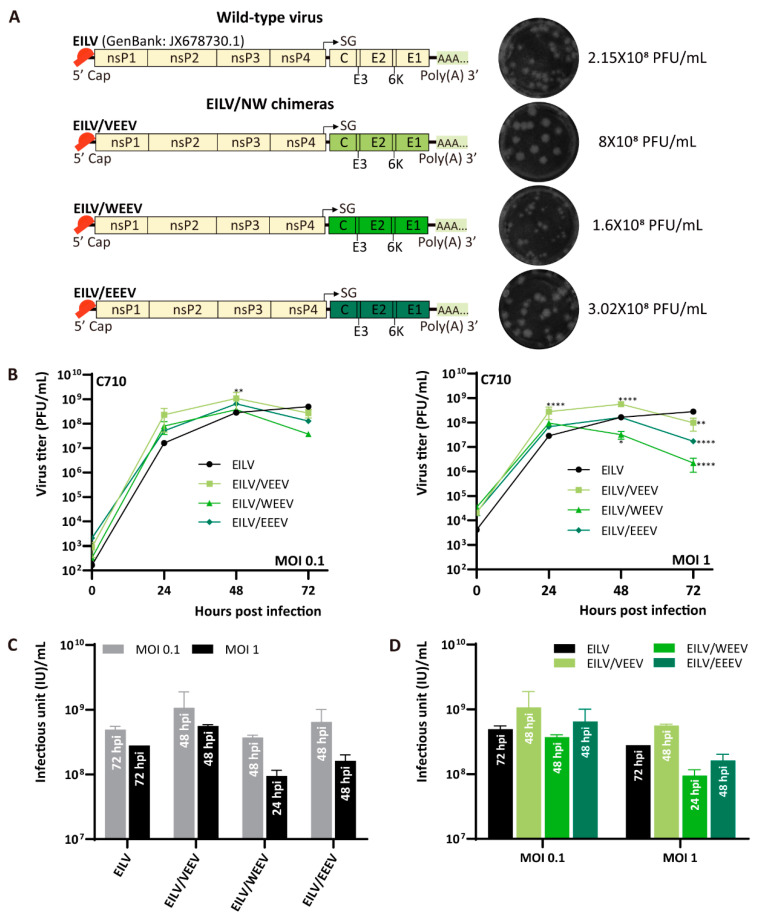
Genome organization and replication kinetics of EILV/ New World (NW) chimeras. (**A**) Genome organization of EILV/NW chimeras. Viruses were rescued on C7/10 cells and harvested at 2 dpe. Rescue titers and plaque phenotypes are displayed. (**B**) Replication of EILV/NW chimeras in C7/10 cells. Cells were infected at an MOI of 0.1 or 1. Media were collected at the indicated time points post-infection, and titers were determined by plaque assay. (**C**) The optimal titers obtained by each virus with different MOIs. (**D**) Comparison of the optimal titers produced by EILV and EILV/OW chimeras. The annotation embedded in each bar in (**C**) and (**D**) indicates the time point of reaching the highest titer. Each sample was titrated twice (*n* = 2). Average titers ±SD (error bars) are shown. **, *p* < 0.01; ****, *p* < 0.0001.

A series of EILV-based chimeras was engineered to express the structural proteins of either Old World (OW) or New World (NW) alphaviruses. The OW and NW viruses have evolved independently in different hemispheres for the last few thousand years. As a result, they reprogrammed their genes for replication in particular species of hosts and developed distinct mechanisms of interfering with the cellular antiviral response [26,27]. The OW viruses used in the present study include SINV strain Toto 1101, MAYV strain Acre27, ONNV strain SG650, and CHIKV strain Caribbean (Figure 1A). The NW viruses involved are VEEV strain TC83, WEEV strain McMillan, and EEEV strain FL93-939 (Figure 2A). The plasmids containing the cDNA clones of these chimeras were constructed and transcribed into capped RNAs in an in vitro system, followed by electroporation into C7/10 cells. Rescued viruses were collected at 48 hpe and titrated on C7/10 cells by performing plaque assay. All chimeras formed clear plaques on C7/10 monolayers and produced similar or higher titers than wild-type EILV, given the titer of EILV was 2.15 × 10^8^ plaque-forming unit (PFU)/mL (Figure 1A and Figure 2A). Amongst the OW chimeras, EILV/CHIKV, EILV/MAYV, and EILV/ONNV yielded titers exceeding 10^9^ PFU/mL, with EILV/CHIKV reached the highest, which was 4.45 × 10^9^ PFU/mL. The titer of EILV/SINV was relatively low (4.57 × 10^8^ PFU/mL) but comparable to that of EILV. In the cluster of NW chimeras, EILV/VEEV yielded the highest titer (8 × 10^8^ PFU/mL). Interestingly, EILV/EEEV produced a titer (3.02 × 10^8^ PFU/mL) similar to that of EILV, while in a previous study [20], the same EILV/EEEV construct yielded a rescue titer two orders of magnitude lower than EILV. Besides, it is worth noting that despite the similar titers, the plaques formed by EILV/WEEV were of smaller size compared with EILV and other chimeras, suggesting a relatively poor replication competency of EILV/WEEV.

### 3.2. Comparative Analysis of Replication of EILV-Based Chimeras

EILV and EILV-based chimeras cannot infect vertebrate cell lines but can exclusively replicate in mosquito cells [12,14,16,20]. To compare the replication kinetics of wild-type EILV and EILV-based chimeras, C7/10 cells were inoculated with viruses with an MOI of 0.1 or 1, respectively. Cell supernatants were taken at 0, 24, 48, and 72 h post-infection (hpi) and titrated on C7/10 cells by plaque assay. Each constructed chimera displayed similar growth curves at these two different MOIs except EILV/WEEV (Figure 1B and Figure 2B). The titer of EILV/SINV, EILV/MAYV, EILV/ONNV, EILV/VEEV, and EILV/EEEV rose by 48 hpi and declined by 72 hpi. EILV/CHIKV rapidly replicated to a high titer at 24 hpi, followed by dramatic descent. EILV/WEEV reached the highest titer by 48 hpi with MOI 0.1, whereas in the case of MOI 1, the titer peaked at 24 hpi and then dropped. Despite the similar replication kinetics with two different MOIs, lower MOI was favorable to produce a higher titer of chimeras (Figure 1C and Figure 2C). It is also interesting to observe that in some cases, the chimeras yielded significantly higher titers than the parental EILV (Figure 1D and Figure 2D). For instance, EILV/CHIKV produced a highest titer 7- or 2.6-fold that of the parental EILV, and EILV/VEEV yielded a highest titer 2-times that of EILV inoculating with either MOIs of 0.1 or 1.

**Figure 3:**
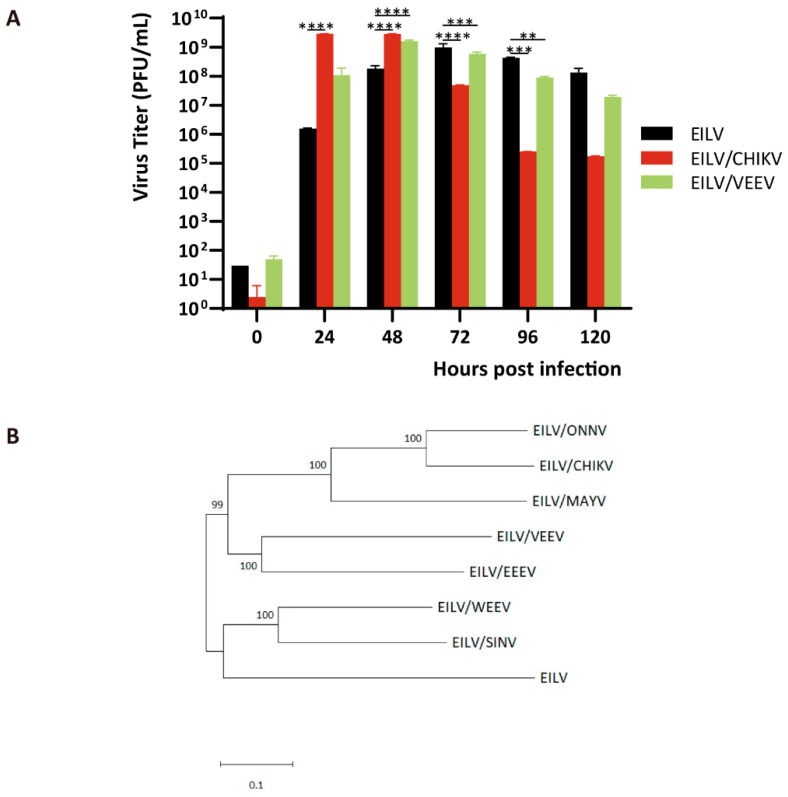
The discrepancy of wild-type EILV and different EILV-based chimeras. (**A**) Persistent replication of EILV, EILV/ Chikungunya virus (CHIKV), and EILV/ Venezuelan equine encephalitis virus (VEEV). Cells were infected with an MOI of 0.01. Media were collected at the indicated time points post-infection, and titers were determined by plaque assay. Each sample was titrated twice (*n* = 2). Average titers ± standard deviations (SD) (error bars) are shown. **, *p* < 0.01; ***, *p* < 0.001; ****, *p* < 0.0001. (**B**) Phylogenetic tree based on the structural sequences of indicated viruses. The phylogenetic tree was inferred based on ClustalW alignment and using the neighbor-joining method. The tree was rooted to the midpoint. The sequence information of chimeras is available upon request.

Notably, unlike the chimeras, the titer of EILV rose continuously by 72 hpi. The descending titers of constructed chimeras by 72 hpi indicated the impaired stability compared with the parental virus. For further evaluation, C7/10 cells were inoculated with EILV/CHIKV, EILV/VEEV, and EILV, respectively, at an MOI of 0.01. Samples were collected every 24 h for 120 h and titrated on C7/10 cells (Figure 3A). The titer of EILV/CHIKV rapidly peaked at 24 and 48 hpi and declined 58-fold by 72 hpi and four orders-of-magnitude by 96 hpi. The release of EILV/VEEV was slower, reaching the highest titer at 48 hpi. The titer then dropped gradually, descending 81-fold by 120 hpi. By contrast, the titer of parental EILV was maximum at 72 hpi, followed by a slight decline, with a drop of 6-fold at 120 hpi. Therefore, in this persistent replication study, EILV/VEEV maintained a more stable status than EILV/CHIKV, although both EILV/VEEV and EILV/CHIKV displayed inferior stability to the parental EILV. This difference in stability results from the intrinsic discrepancy between EILV/CHIKV and EILV/VEEV. According to the phylogenetic tree generated using the structural sequences of constructed chimeras (Figure 3B), EILV has proximity to EILV/WEEV and EILV/SINV. Given the evolutionary track that WEEV is a recombinant virus with glycoproteins from a SIN-like virus and the rest of the sequence from an EEE-like virus [1,28], EILV/WEEV has a resemblance to EILV/SINV and EILV/EEEV. Moreover, EILV/EEEV and EILV/VEEV are shown to be close relatives in the phylogenetic tree (Figure 3B). Therefore, it can be inferred that EILV is most likely to incline to the chimeras whose structural genes are from NW alphaviruses.

### 3.3. Generation of EILV-Based Double Subgenomic Vector Expressing ZIKV NS1 Protein

**Figure 4:**
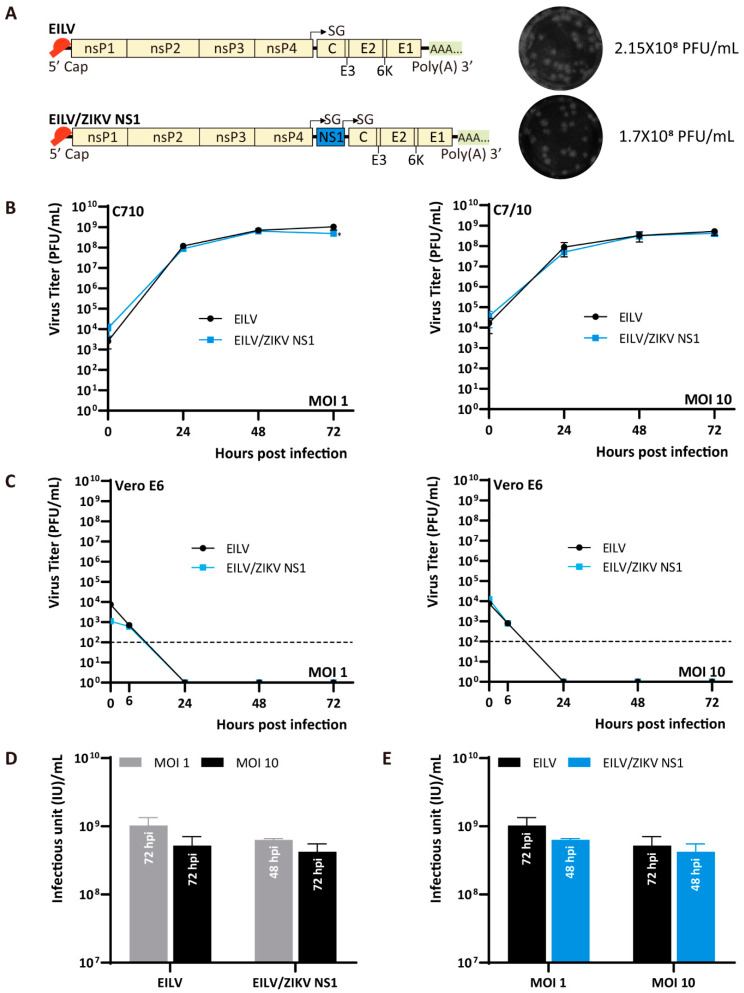
Genome organization and replication kinetics of EILV-based double subgenomic (SG) variant expressing Zika virus (ZIKV) nonstructural 1 (NS1) protein. (**A**) Genome organization of EILV/ NS1 recombinant. Virus was rescued on C7/10 cells and harvested at 3 dpe. Rescue titers and plaque phenotypes are displayed. (**B**) Replication kinetics in C7/10 and Vero E6 cells. C7/10 or Vero E6 cells were infected at an MOI of 1 or 10. Media were collected at the indicated time points post-infection, and titers were determined by plaque assay. (**C**) The optimal titers obtained by each virus with different MOIs. (**D**) Comparison of the optimal titers produced by EILV and EILV/NS1. The annotation embedded in each bar in (**D**) and (**E**) indicates the time point of reaching the highest titer. Each sample was titrated twice (*n* = 2). Average titers ±SD (error bars) are shown. *, *p* < 0.05.

As the feasibility of exploiting the EILV-based double SG vector to produce authentic antigens has not been investigated, in the present study, EILV-based double SG vectors expressing antigens of varied sizes were constructed and characterized. Firstly, ZIKV non-structural 1 (NS1) protein containing a signal peptide was adopted, the length of which is 1188 bp. This glycoprotein plays a crucial role in viral RNA replication and immune evasion [29] and is a promising target for vaccine development [30,31]. The resulting recombinant virus was readily rescued on C7/10 cells and displayed a similar titer to the parental virus (Figure 4A). The plaques formed by EILV/NS1 and wild-type EILV were alike (Figure 4A). The replication kinetics were evaluated at MOI 1 and 10. On C7/10 cells, the double SG EILV/NS1 variant exhibited a growth curve almost indistinguishable from that of the parental EILV at either MOI 1 or 10 (Figure 4B). It reached a high titer of 6.29 × 10^8^ PFU/mL at MOI 1 and a titer of 4.24 × 10^8^ PFU/mL at MOI 10. The maximum titers obtained with different MOIs were comparable (Figure 4D). The highest titers produced by the recombinant and the parental viruses were also similar (Figure 4E). Consistent with previous studies [12,14], both EILV/NS1 and EILV cannot replicate in vertebrate cells (Figure 4C).

### 3.4. Efficient Expression of ASFV Antigens in the Original Mosquito Cells by EILV-Based Double SG Vectors

**Figure 5:**
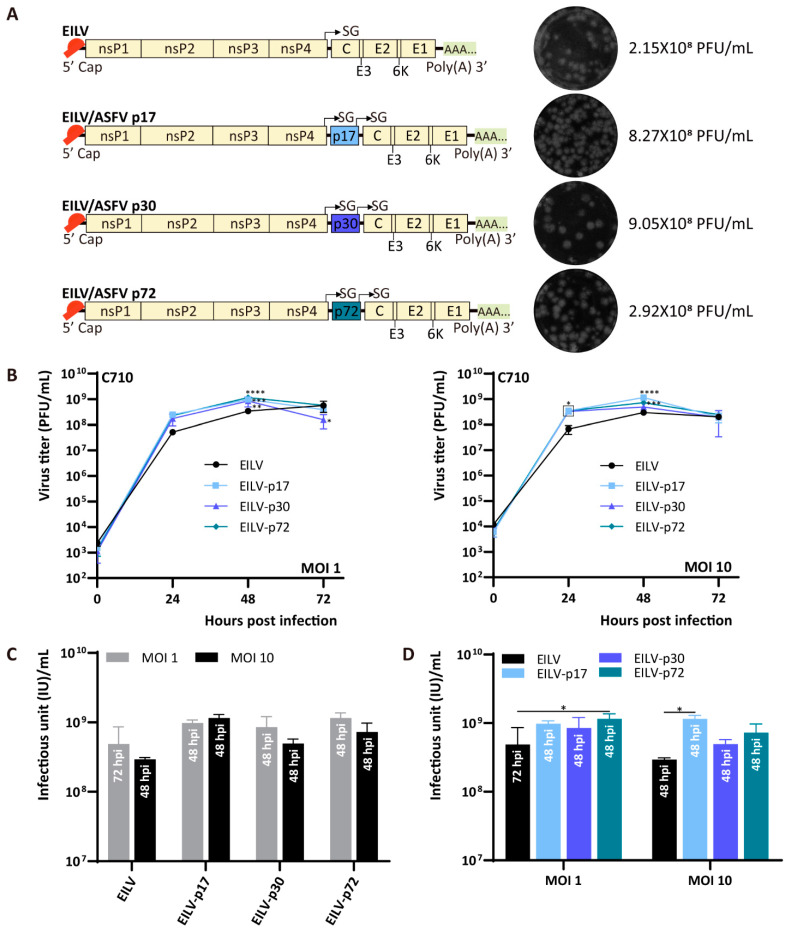
Genome organization and replication kinetics of EILV-based double SG variants expressing African swine fever virus (ASFV) antigens. (**A**) Genome organization of EILV/p17, EILV/p30, and EILV/p72 recombinants. Viruses were rescued on C7/10 cells and harvested at 3 dpe. Rescue titers and plaque phenotypes are displayed. (**B**) Replication kinetics in C7/10 cells. Cells were infected at an MOI of 1 or 10. Media were collected at the indicated time points post-infection, and titers were determined by plaque assay. (**C**) The optimal titers obtained from each virus with different MOIs. (**D**) Comparison of the optimal titers produced by EILV and recombinants. The annotation embedded in each bar in (**C,D**) indicates the time point reaching the highest titer. Each sample was titrated twice (*n* = 2). Average titers ± standard deviations SD (error bars) are shown. *, *p* < 0.05; **, *p* < 0.01; ***, *p* < 0.001; ****, *p* < 0.0001.

ASFV is the causative pathogen of the devastating African swine fever (ASF) that can kill almost all the infected pigs, posing a threat to global pork production and food security [32]. Currently, there are no vaccines available to prevent ASF. Therefore, it is of high priority to develop potential diagnostic tools and vaccines to control and halt this disease. By applying the antigens of this far-distant veterinary swine viral pathogen in a size-dependent manner, we validated the capability of EILV to incorporate foreign materials regardless of pathogen types and gene length. Three ASFV antigens were introduced individually, including major capsid protein p72, inner membrane protein p17, and early structural protein p30 [33], the gene lengths of which are 1941, 354, and 606 bp, respectively. All the constructed double SG variants were readily rescued (Figure 5A). Specifically, EILV/p17 and EILV/p30 yielded higher titers than the original EILV, reaching 8.27 × 10^8^ and 9.05 × 10^8^ PFU/mL, respectively. The titer produced by EILV/p72 was comparable to that of EILV (2.92 × 10^8^ PFU/mL). However, despite the original titer difference, three double SG vectors displayed similar growth curves on C7/10 cells with an MOI of either 1 or 10 (Figure 5B). These vectors displayed titers 2–4-fold higher than the parental EILV at 24 hpi and reached the highest titers at 48 hpi, ranging from 4.92 × 10^8^ to 1.15 × 10^9^ PFU/mL and significantly exceeding the titers of EILV. The titers then slightly declined by 72 hpi. Two MOIs had no significant effect on the optimal titers obtained by the three double SG variants (Figure 5C). Notably, either at MOI 1 or 10, the highest titers of the double SG recombinants were close to or even higher than that of EILV (Figure 5D). Conclusively, these double SG constructs, especially the EILV/p72 construct incorporating a large insert of about 2 kb, displayed the EILV-based expression platform’s superior tolerability.

**Figure 6:**
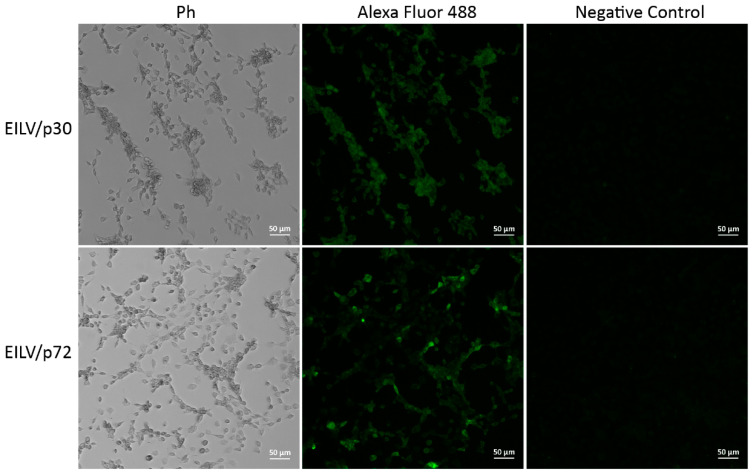
Expression of ASFV p30 and p72 antigens in C7/10 cells infected with EILV/p30 and EILV/p72 recombinants. Cells were inoculated with viruses with an MOI of 5. IFA was performed at 24 hpi.

The antigenicity of the expressed heterogeneous proteins was subsequently determined by IFA. C7/10 cells were infected with EILV/p72 or EILV/p30 variant with an MOI of 5 and fixed and stained at 24 hpi. The fluorescence was readily observed in infected cells (Figure 6), indicating that the target antigens, ASFV p72 and p30, were produced and recognized by antigen-specific monoclonal antibodies. The expression of ASFV p17 has not been verified due to the lack of commercially available antibodies. These produced antigens can be applied to the serological diagnosis of ASV [32].

### 3.5. Development of a Novel EILV-Based Vaccine Candidate against ZIKV and CHIKV

**Figure 7:**
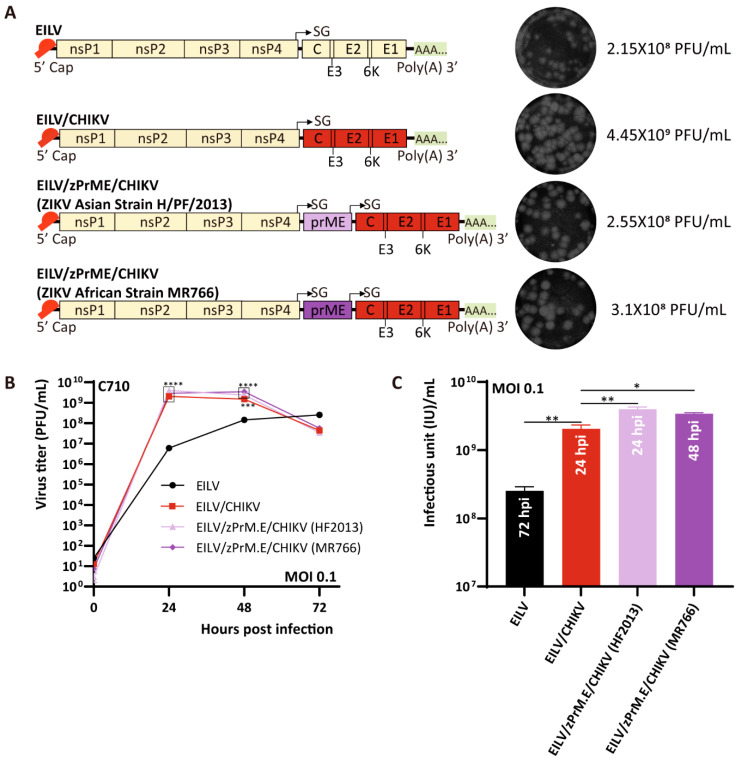
Genome organization and replication kinetics of EILV-based chimeric double SG variants expressing ZIKV and CHIKV antigens. (**A**) Genome organization of EILV/ ZIKV pre-membrane and envelope proteins (zPrME) /CHIKV recombinants. Viruses were rescued on C7/10 cells and harvested at 3 dpe. Rescue titers and plaque phenotypes are displayed. (**B**) Replication kinetics in C7/10 cells. Cells were infected at an MOI of 0.1. Media were collected at the indicated time points post infection, and titers were determined by plaque assay. (**C**) Comparison of the optimal titers produced by the recombinants and parental viruses. The annotation embedded in each bar in (**C**) indicates the time point reaching the highest titer. Each sample was titrated twice (*n* = 2). Average titers ±SD (error bars) are shown. *, *p* < 0.05; **, *p* < 0.01; ***, *p* < 0.001; ****, *p* < 0.0001.

In a preliminary study, chimeric EILV/VEEV-based double SG vectors expressing GFP (720 bp in length) or ZIKV NS1 (1188 bp in length) were generated and characterized (Figure S1). Given the fact that ZIKV and CHIKV can be transmitted by the same mosquito vectors and co-circulate in many parts of the world, bivalent vaccines against both ZIKV and CHIKV infections have been developed, which were based on a vaccinia virus vector [9,10] or a vesicular stomatitis virus vector [11]. A novel, multivalent vaccine candidate utilizing the EILV-based chimeric double SG platform was generated in the current study. EILV/CHIKV chimera encompassing the structural protein cassette of CHIKV has been constructed and can reach a titer as high as 10^9^ PFU/mL (Figure 1) [16,19]. On this basis, the gene sequences encoding the structural proteins of ZIKV (prME, 2016 bp in length) were inserted by introducing a second EILV SG promoter downstream of the prME coding region (Figure 7A). Two prME sequences, derived from either Asian strain H/PF/2013 or African strain MR766, were adopted. The resulting constructs, EILV/ ZIKV prME (zPrME) /CHIKV H/PF/2013 and MR766, were rescued on C7/10 cells and yielded titers comparable to that of wild-type EILV but one order of magnitude lower than that of the parental EILV/CHIKV (Figure 7A). The plaques formed by these two chimeric double SG vectors were similar to those produced by the parental chimera (Figure 7A). The replication kinetics was evaluated on C7/10 cells with an MOI of 0.1. The growth curves of EILV/zPrME/CHIKV variants were indistinguishable from that of EILV/CHIKV (Figure 7B), indicating that the large inserts did not impair the stability of parental chimera. Like EILV/CHIKV, the EILV/zPrME/CHIKV recombinants rapidly reached high titers at 24 hpi and 48 hpi. The maximum titers of these two recombinants were one order of magnitude higher than that of EILV and even exceeded that of EILV/CHIKV (Figure 7C).

**Figure 8:**
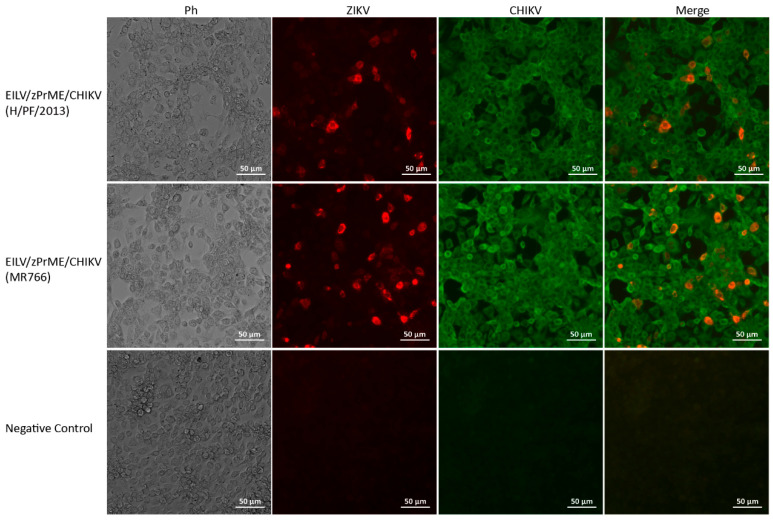
Expression of ZIKV and CHIKV antigens in C7/10 cells infected with EILV/zPrME/CHIKV recombinants. Cells were inoculated with viruses with an MOI of 5. IFA was performed at 24 hpi. The envelope (E) protein of ZIKV was labeled with red fluorescence, and the CHIKV antigens were labeled with green fluorescence.

The antigenicity of the expressed ZIKV and CHIKV antigens was also determined by IFA on C7/10 cells. The cells were inoculated with EILV/zPrME/CHIKV H/PF/2013 or MR766 recombinant with an MOI of 5 and fixed and stained at 24 hpi. The E protein of ZIKV was labeled with red fluorescence, and the CHIKV antigens were labeled with green fluorescence. Both types of fluorescence were observed in infected cells and displayed overlay in the merged images (Figure 8), indicating that the cells expressed ZIKV and CHIKV antigens simultaneously. There are two scenarios for the generated antigens. The ZIKV envelope (E) proteins may integrate into the EILV/CHIKV chimeric viruses. The resulting viral particles can initiate single-round infection in vivo and activate the immune system. Alternatively, the produced ZIKV E and M proteins may form subviral particles, and therefore, the bivalent vaccines can consist of EILV/CHIKV chimeras and ZIKV virus-like particles. A thorough validation is required in the future.

## 4. Discussion

Owing to the ability to mediate highly efficient gene expression in the cytoplasm without disturbing the host genomic DNA, combined with the availability of cDNA clones and the genetic flexibility, alphaviruses have been extensively investigated as sophisticated platforms for high-level expression of heterologous genes [6,34]. These alphavirus-based platforms can be utilized as vaccine candidates [35] and diagnostic tools [19], as well as functional experimental tools [36]. EILV is a unique alphavirus that exclusively propagates in mosquito cells and is noninfectious for vertebrates [14]. This host-restricted character additionally grants EILV-based expression vectors a favorable safety profile compared with other alphavirus-based platforms [16,19,20].

By replacing the structural genes of EILV with those of different OW and NW alphaviruses, we generated a series of chimeric recombinants. The OW and NW viruses have evolved independently for the last few thousand years and exhibit significant divergence in the genomes. Our data proved that the EILV genome was compatible with these genetically distant lineages of alphaviruses (Figure 1A and Figure 2A). However, the constructed chimeras and the parental EILV displayed some variations. In the term of plaque phenotype, most chimeras somewhat exhibited heterogeneity in plaque morphology, suggesting potential for additional adaptations for efficient replication [37]. The plaques produced by EILV/SINV were most similar to those of EILV. EILV/VEEV generated significantly larger plaques than EILV, indicating increased infectiousness, while EILV/WEEV formed smaller plaques than EILV and other chimeras.

In the aspect of growth kinetics (Figure 1B and Figure 2B), most chimeras can replicate to a higher titer and release progeny virions at a faster rate than the parental EILV. This variation in replication may result from several factors, including the interaction between the heterologous capsid protein and the chimeric genome for packaging and delivery of the genome to the ribosomes, followed by the translation of the genome. Despite the increased replication rate in the early stage of infection, the titers of all chimeras declined by 72 hpi while the titer of EILV rose continuously, indicating the chimeras’ impaired stability. Therefore, we selected EILV/CHIKV and EILV/VEEV as the representatives of EILV/OW and EILV/NW constructs, respectively, and compared their stability with the parental EILV in a persistent replication study. Although EILV/CHIKV and EILV/VEEV exhibited reduced stability compared with EILV, EILV/VEEV maintained a more stable status than EILV/CHIKV during persistent infection (Figure 3A). The reason for this difference in stability can be deduced from the phylogenetic relationship between EILV and the generated chimeras (Figure 3B). EILV displays proximity to EILV/WEEV and EILV/SINV. As WEEV is a recombinant virus derived from a SIN-like virus and an EEE-like virus [1,28], EILV/WEEV somewhat resembles EILV/SINV and EILV/EEEV. In addition, EILV/EEEV and EILV/VEEV are phylogenetically close synthetic variants. Therefore, it can be inferred that EILV is most likely to incline to the chimeras whose structural genes are from NW viruses.

EILV-based chimeras are confined to expressing alphavirus-derived antigens. To produce antigens of other origins, we introduced a second SG promoter into the EILV genome or the EILV/CHIKV chimera to construct replication-competent double SG vectors. The resulting variants were readily rescued in mosquito cells and exhibited similar replication kinetics to the parental virus (Figure 4 and Figure 5). The antigenicity of expressed antigens was validated by binding with specific monoclonal antibodies (Figure 6 and Figure 8). All tested antigens in the current study are derived from arboviruses. Due to the host-restricted character of EILV, these double SG vectors are confined to infect mosquito cells. This can be a drawback of the EILV-based vectors. Since there is an inherent discrepancy between insect and mammalian protein processing pathways [38], mosquito cells may fail to accurately confer post-translational modifications to some humanized antigens.

The size of inserted transgenes has a substantial impact on the retention of gene expression. It was reported that the large firefly luciferase (1650 bp in length) was lost rapidly from SINV- or VEEV-based replication-competent vectors, while the smaller nanoLuc (513 bp in length) exhibited better stability [21]. In our current study, the gene length of ASFV p72 is 1941 bp, and the sequence of ZIKV prME is as long as 2016 bp. The expression of these genes may decline quickly during infection. Infecting cells with a high MOI and harvesting target proteins at an early stage may increase the yields. It is also crucial to investigate whether the encoded antigens can be incorporated into the alphavirus particles. In the case of ASFV antigen production, determining whether the proteins are expressed extracellularly or intracellularly is essential to choosing the appropriate antigen purification method. For the chimeric double SG vaccine candidates, the expression of ZIKV prME genes may result in the generation of immunogenic subviral particles consisting of E and M proteins, or possibly, the E protein can integrate into the EILV/CHIKV chimeric virions. More detailed studies are required to address these questions. Moreover, the diagnosis application of ASFV-related double SG vectors needs to be examined in relation to sensitivity, specificity, stability, and reproducibility. The efficacy of newly conceived bivalent vaccine constructs will be validated with an in vivo study.

Lastly, our data demonstrated the productive replication and heterologous protein expression of EILV-based variants in the original mosquito cells. Large scale production can be facilitated by modulating MOI and harvesting at relevant time points. Also, as the chimeric double SG vectors can reach higher titers than wild-type EILV and, predictably, EILV-based double SG variants, it would be intriguing to develop chimeric double SG recombinants to produce heterologous antigens in vitro.

## 5. Conclusions

In summary, our study proved that the EILV genome is highly tolerant to the structural genes of other alphaviruses and the inserted additional SG promoters. Given this outstanding compatibility and versatility, EILV-based expression platforms hold great promise to be applied to the development of various vaccines and diagnostic tools.

## Figures and Tables

**Table 1:** Antibodies used for immunofluorescence assay (IFA).

Name	Citation	Supplier	Cat No.	Clone No.
Anti-ASFV p30 (mouse)	Figure 6	Creative Biolabs	MOB-Z054-YC	DEC.YC054
Anti-ASFV p72 (mouse)	Figure 6	Creative Biolabs	MOB-Z055-YC	DEC.YC055
Anti-ZIKV E protein (rabbit)	Figure 8	Sino Biological	40543-R029	
Anti-CHIKV	Figure 8	Invitrogen	MA5-18180	D51Q
Goat anti-rabbit IgG (H + L), Alexa Fluor 488	Figure 8	Invitrogen	A-11008	
Donkey anti-mouse IgG (H + L), Alexa Fluor 488	Figure 6 and Figure 8	Invitrogen	A-21202	
Goat anti-rabbit IgG (H + L), Alexa Fluor 594	Figure 8	Invitrogen	A-11012	

(Creative Biolabs, New York, USA; Sino Biological, Beijing, China; Invitrogen, Waltham, MA, USA).

## Data Availability

Not applicable.

## Supplementary Materials

The following are available online at https://www.mdpi.com/article/10.3390/v13040660/s1, Figure S1: Genome organization and replication kinetics of double SG variants based on EILV/VEEV chimera.
