# Supplementary figures and images for: A Productive Expression Platform Derived from Host-Restricted Eilat Virus: Its Extensive Validation and Novel Strategy
Source: Viruses. 2021 Apr 11;13(4):660. doi: 10.3390/v13040660 (PMC8069092; doi:10.3390/v13040660)

**A**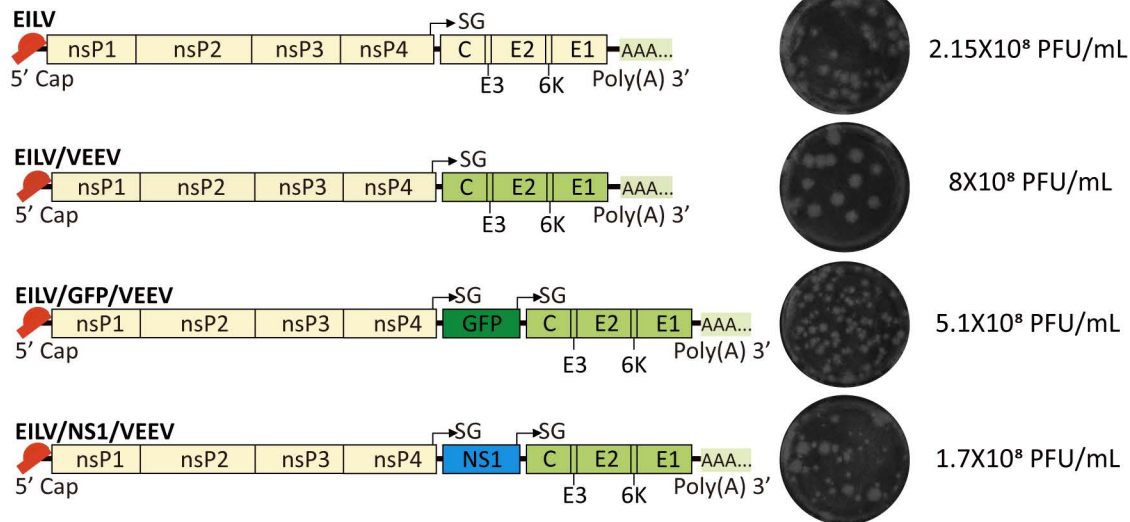**B**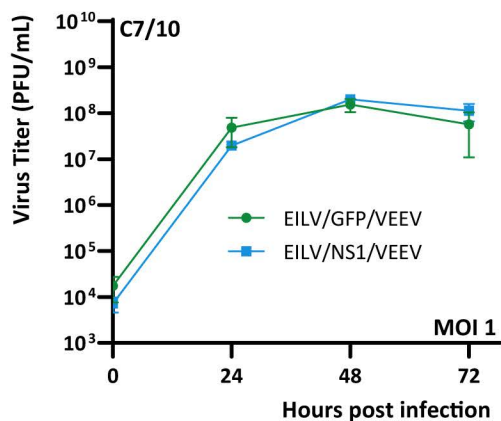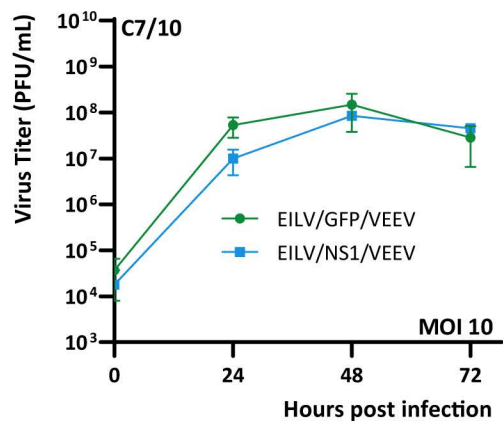

Supplement: Supplementary file 1 [file viruses-13-00660-s001.pdf]
